# Chronic stress, cortisol dysregulation, and neurodegenerative vulnerability: mechanistic pathways linking HPA-axis dysfunction to Alzheimer’s disease risk

**DOI:** 10.3389/fnagi.2026.1883880

**Published:** 2026-07-02

**Authors:** Danah F. Almalki, Rawiyah A. Alkabkabi, Raghad A. Wayyani, Abdulrahman S. Alharthi, Hala A. Aljuhani, Razan M. Mahyub, Hadeel S. Bakhsh, Afnan M. Alomani, Nouf N. Almhmadi, Ahases A. Aljohani, Abdullah A. Tawakul

**Affiliations:** 1 Faculty of Medicine, Umm Al-Qura University, Makkah, Saudi Arabia; 2 General Medicine Practice Program, Batterjee Medical College, Jeddah, Saudi Arabia; 3 Department of Medicine, Faculty of Medicine, Umm Al-Qura University, Makkah, Saudi Arabia

**Keywords:** allostatic load, Alzheimer’s disease, glucocorticoids, HPA axis, neurodegeneration, neuroinflammation, synaptic plasticity

## Abstract

Chronic psychological stress is increasingly recognized as a silent risk factor of long-term brain vulnerability and a potential modifier of neurodegenerative disease trajectories. The persistent activation of the hypothalamic–pituitary–adrenal (HPA) axis and the consequent dysregulation of cortisol exert extensive influences on neural, immune, and metabolic pathways linked to cognitive decline and dementia. This review synthesizes current mechanistic and clinical evidence concerning the effects of chronic stress-induced HPA-axis dysfunction on neurodegenerative susceptibility and Alzheimer’s disease risk. A structured narrative synthesis was conducted using literature from PubMed/MEDLINE, Scopus, and Web of Science, integrating multidisciplinary evidence from established biological and mechanistic domains. Present evidence suggests that prolonged exposure to glucocorticoids is associated with structural and functional alterations in the brain, including hippocampal atrophy, prefrontal cortical dysfunction, reduced synaptic plasticity, and increased amygdala activity. Chronic dysregulation of cortisol may result in neuroinflammation, disruption of the blood–brain barrier, induce mitochondrial dysfunction, and impair neuronal integrity. These interconnected mechanisms contribute to amyloid accumulation, tau pathology, and the progressive decline of neural resilience. Rather than serving as the root cause, chronic psychological stress can increase the likelihood of neurodegeneration by triggering complex interactions between the neuroendocrine and neuroimmune systems that accelerate the already existing pathological pathways toward neurodegeneration. Recognizing chronic stress as a potentially modifiable biological risk factor may improve early risk stratification and identification of HPA-axis dysregulation, ultimately driving preventive strategies targeting stress-related neurobiological pathways.

## 1 Introduction

Dementia constitutes one of the most pressing global public-health challenges, with profound human, societal, and economic consequences and a projected rise in prevalence driven by population aging (World Health Organization, 2025). Despite substantial advances in elucidating the molecular and systems-level biology of neurodegeneration, contemporary evidence emphasizes that a meaningful proportion of dementia burden is potentially preventable or delayable through life-course modification of established risk factors, underscoring the need to broaden prevention frameworks beyond traditional clinical determinants (Livingston et al., 2024). In this context, chronic psychological stress has emerged as a plausible, highly prevalent exposure capable of reshaping long-term physiology and brain health through cumulative “wear and tear” mechanisms often conceptualized as allostatic load (McEwen and Stellar, 1993). Seminal work further clarified that stress mediators can be adaptive in the short term yet become pathogenic when activation is prolonged, dysregulated, or repeatedly triggered, thereby promoting systemic and neural vulnerability over time (McEwen, 1998).

Central to the stress response is the hypothalamic–pituitary–adrenal (HPA) axis, which regulates glucocorticoid secretion with strong circadian and ultradian patterning and is normally constrained by negative feedback signaling (Uchoa et al., 2014). Under chronic stress, multiple phenotypes of HPA dysregulation may occur including sustained hypercortisolemia, a flattened diurnal slope, and impaired feedback inhibition. Each of these having potential relevance to neurocognitive trajectories. Accordingly, we are seeing a growing research emphasis on biomarkers that capture longer-term glucocorticoid exposure to better index cumulative stress biology across weeks to months, complementing short-window measures such as salivary or plasma cortisol (Stalder et al., 2016).

At the brain level, converging human evidence links chronic glucocorticoid elevation to structural and functional changes in regions responsible for learning, memory, and the onset of neurodegeneration, particularly the hippocampus and connected cortical networks. Classic longitudinal work demonstrated that cortisol levels across aging can predict hippocampal atrophy and memory decline (Lupien et al., 1998). More recent multimodal imaging studies across the Alzheimer’s disease (AD) spectrum report associations between higher plasma cortisol and reduced cerebral glucose metabolism (FDG hypometabolism) as well as lower gray-matter volume in regions implicated early in AD pathology, suggesting a measurable pathway connecting HPA-axis dysregulation to neurodegenerative signatures (Wirth et al., 2019). Complementing these mechanistic observations, prospective epidemiologic data using repeated long-term cortisol assessments indicate that greater cumulative glucocorticoid exposure may predict subsequent AD risk (Ennis et al., 2017). Systematic reviews and meta-analyses further support the presence of cortisol hypersecretion, mostly evident in morning measures, in AD compared with cognitively normal controls, while also highlighting heterogeneity in study designs, confounding structures, and the possibility of reverse causality as neurodegeneration itself may perturb HPA regulation (Zheng et al., 2020).

Notably, the physiological pathways mediating the effects of chronic stress intersect with the aging process. In fact, age-related changes in HPA-axis feedback sensitivity, neuroimmune regulation, and neuronal reserve mean that older adults are uniquely vulnerable to the cumulative neurobiological consequences of sustained stress exposure (McEwen, 2007; Lupien et al., 1998). Importantly, positioning stress-cortisol biology within dementia frameworks should not be limited to direct “hormonal toxicity” models. Neurodegeneration unfolds through tightly coupled interactions among synaptic dysfunction, proteinopathy (amyloid and tau), vascular integrity, and neuroimmune activation. Therefore, modern reviews argue that chronic stress and glucocorticoids can act as pathway amplifiers to accelerate vulnerability and brain disease progression by worsening existing brain pathologies such as amyloid/tau dynamics and inflammatory signaling. Rather than serving as isolated risk factors, they intersect with biological disease pathways to speed up the progression of AD and similar conditions (Burke et al., 2024). Building on this integrative insight, this review aims to consolidate evidence linking chronic stress exposure to HPA-axis dysregulation and downstream brain and neuroimmune changes relevant to dementia. In addition to identifying key methodological gaps and emphasizing translational priorities for prevention and intervention research.

## 2 Methods

### 2.1 Review design

This study was conducted as a structured narrative review aimed at integrating multidisciplinary evidence examining the relationship between chronic psychological stress, cortisol dysregulation, and dementia risk. PRISMA-based screening, risk-of-bias scoring, and meta-analysis were not performed given the methodological heterogeneity across neuroendocrinology, psychiatry, neuroimaging, immunology, and longitudinal epidemiology. Instead, a narrative synthesis approach was selected to enable conceptual and mechanistic integration across diverse study designs. Although not conducted as a formal systematic review, predefined search strategies, explicit eligibility criteria, and structured thematic domains were applied to enhance methodological transparency, reproducibility, and analytical rigor.

### 2.2 Literature search strategy

A comprehensive literature search was performed using PubMed/MEDLINE, Scopus, and Web of Science. The search included all eligible studies published from database inception through January 2026. Reference lists of relevant reviews and primary research articles were manually screened to identify additional studies not captured in the initial search.

Search terms were constructed using both controlled vocabulary (MeSH terms in PubMed) and free-text keywords to maximize sensitivity. Core conceptual domains included chronic stress, HPA-axis dysfunction, cortisol dysregulation, neuroinflammation, structural brain alterations, cognitive decline, and dementia.

#### 2.2.1 PubMed Boolean search strategy

The primary PubMed search string was structured as follows:

(“chronic stress” OR “psychological stress” OR “anxiety disorders”) AND (“HPA axis” OR “hypothalamic–pituitary–adrenal axis” OR “cortisol dysregulation” OR “glucocorticoids”).

AND (“hippocampal atrophy” OR “prefrontal cortex” OR “neuroinflammation” OR “immune dysregulation”).

AND (“cognitive decline” OR “mild cognitive impairment” OR “dementia” OR “Alzheimer’s disease”).

Medical Subject Headings (MeSH) were incorporated where applicable to enhance search precision, and Boolean operators (AND/OR) were systematically applied to optimize retrieval sensitivity and specificity. Search terms were iteratively refined to ensure comprehensive capture of mechanistic, neurobiological, and longitudinal evidence.

### 2.3 Eligibility criteria

Studies were eligible for inclusion if they were peer-reviewed articles published in English and addressed at least one of the following domains: chronic psychological stress or anxiety-related conditions; assessment of cortisol levels or HPA-axis function; structural or functional brain alterations; neuroinflammatory mechanisms; or cognitive decline and dementia outcomes.

Eligible study designs included cross-sectional analyses, prospective cohort studies, longitudinal investigations, neuroimaging studies, and experimental animal models elucidating biological mechanisms relevant to neurodegeneration. Experimental studies were included when they provided mechanistic insights linking glucocorticoid dysregulation to structural or molecular changes associated with neurodegenerative processes.

Studies focusing exclusively on acute stress responses without relevance to chronic exposure were excluded. Case reports without broader mechanistic implications, editorials, commentaries, conference abstracts, and non-peer-reviewed literature were also excluded.

### 2.4 Study selection process

Titles and abstracts identified through the search strategy were screened for conceptual relevance to chronic stress-related endocrine dysregulation and its neurobiological consequences. Full-text articles of potentially eligible studies were subsequently reviewed in detail to determine inclusion.

When multiple studies addressed similar research questions, preference was given to those with larger sample sizes, longer follow-up durations, replication across independent cohorts, and robust methodological design. Studies were critically appraised for internal validity, measurement reliability, and biological plausibility. Interpretive emphasis was placed on replicated findings and converging lines of mechanistic evidence. The sections in our paper were reviewed independently by the authors to ensure all included sources directly addressed the molecular pathways under discussion. Studies meeting the inclusion criteria were incorporated into the narrative synthesis.

### 2.5 Data extraction

For each included study, relevant methodological and outcome variables were extracted, including study design, population characteristics, stress assessment methodology, cortisol measurement modality (serum, salivary, diurnal slope, or hair cortisol), neurobiological endpoints (structural imaging findings, functional alterations, inflammatory markers), cognitive outcomes, dementia incidence where applicable, and reported study limitations.

Data extraction focused particularly on identifying mechanistic pathways linking chronic HPA-axis dysregulation to structural brain vulnerability, neuroimmune activation, and progressive cognitive impairment.

### 2.6 Thematic synthesis approach

Findings were synthesized narratively and organized into predefined mechanistic domains reflecting the biological progression from chronic stress exposure to neurodegenerative vulnerability. These domains included HPA-axis dysregulation, cortisol-induced structural and functional brain alterations, neuroimmune interactions and inflammatory amplification, AD-related molecular cascades, and longitudinal associations between cortisol dysregulation and dementia risk.

An integrative conceptual framework was developed to illustrate the convergence of neuroendocrine and neuroinflammatory pathways contributing to synaptic dysfunction and neurodegeneration. Emphasis was placed on coherence across disciplines and on identifying areas of convergence and uncertainty within the literature.

For terminological consistency, “HPA-axis dysregulation” is used to describe disrupted neuroendocrine stress regulation at the system level, while “cortisol dysregulation” refers specifically to downstream endocrine manifestations arising from this systemic disruption such as hypercortisolemia, flattened diurnal slopes, or blunted reactivity. Concurrently, “HPA-axis dysfunction” is reserved for contexts implying structural or receptor-level impairment.

### 2.7 Quality considerations

The reporting of this narrative review was guided by the SANRA framework (Baethge et al., 2019). Although a formal quantitative risk-of-bias scoring system was not applied, methodological rigor was critically evaluated with attention to sample size, statistical power, follow-up duration in longitudinal studies, validity of cortisol measurement techniques, and consistency of findings across independent cohorts. We took potential confounding variables into consideration, including psychiatric comorbidity, lifestyle factors, and reverse causality in observational designs.

Studies with substantial methodological limitations were interpreted cautiously and contextualized within the broader evidence base. We prioritized results that were consistently reported and supported by established biological mechanisms.

### 2.8 Ethical considerations

This review synthesizes previously published data and does not involve direct interaction with human participants or access to individual-level data. Therefore, institutional ethical approval was not required.

## 3 Neuroendocrine mechanisms of chronic stress

Chronic psychological stress is biologically embedded through sustained activation of the HPA-axis, a central neuroendocrine system that coordinates short-term adaptation but may become maladaptive under prolonged demand. Over time, persistent stress exposure can shift the HPA-axis from a tightly regulated, time-structured response into a dysregulated state characterized by altered cortisol dynamics, impaired feedback inhibition, and downstream effects on stress-sensitive neural circuits. These neuroendocrine changes provide a mechanistic substrate through which chronic stress may increase vulnerability to cognitive and neurodegenerative outcomes, particularly when coupled with immune and metabolic dysregulation (Mbiydzenyuy and Qulu, 2024; Gutierrez Nunez et al., 2025; Herman et al., 2016).

### 3.1 HPA-axis physiology and feedback regulation

The HPA-axis coordinates the endocrine stress response via hierarchical signaling from the hypothalamus to the pituitary and adrenal cortex. In response to perceived stressors, corticotropin-releasing factor/hormone (CRF/CRH) is synthesized and released from the paraventricular nucleus (PVN) of the hypothalamus and acts on the anterior pituitary to stimulate secretion of adrenocorticotropic hormone (ACTH). ACTH then circulates to the adrenal cortex, particularly the zona fasciculata, driving the synthesis and release of glucocorticoids (cortisol in humans) (Mbiydzenyuy and Qulu, 2024; Herman et al., 2016).

A key feature of HPA regulation is negative feedback, whereby circulating glucocorticoids suppress upstream CRH and ACTH signaling to terminate the response and restore homeostasis. This feedback control is mediated largely by glucocorticoid receptors (GRs) and mineralocorticoid receptors distributed across peripheral tissues and key brain regions involved in stress regulation, including the hippocampus and prefrontal cortex. In acute stress, this framework ensures that glucocorticoid exposure is time-limited and context-appropriate (Gutierrez Nunez et al., 2025; Kadmiel and Cidlowski, 2013). However, with chronic stress exposure, repeated or prolonged activation places sustained demands on the system, increasing the likelihood of feedback inefficiency and altered receptor sensitivity. Such changes can shift the HPA-axis from an adaptive, self-limited response toward dysregulation, including altered set-points, diminished feedback inhibition, and abnormal responsivity to subsequent stressors (Gutierrez Nunez et al., 2025; Herman et al., 2016).

### 3.2 Patterns of cortisol dysregulation

Cortisol secretion is not merely defined by “how much” is produced, but by “when” and “how” it is released. Under normal conditions, cortisol follows a circadian rhythm with superimposed pulsatile (ultradian) secretion, supporting metabolic, immune, and cognitive functions aligned with daily physiology. Acute stress typically induces a transient increase in ACTH and cortisol, followed by a return to baseline once feedback mechanisms take effect (Gutierrez Nunez et al., 2025; Herman et al., 2016). Chronic stress can disrupt these rhythms, leading to clinically and biologically significant dysregulation of cortisol levels (sustained hypercortisolemia). In early or persistent phases of chronic stress, basal cortisol levels may remain elevated or show exaggerated responses to stressors, increasing cumulative glucocorticoid exposure. Prolonged elevations can influence neural plasticity, energy metabolism, and immune signaling, particularly in stress-sensitive brain structures (Gutierrez Nunez et al., 2025; Herman et al., 2016). Beyond elevation, this dysfunction can show up as flattened diurnal rhythm. By disrupting this normal morning peak and evening trough, that implies that the brain and periphery are exposed to cortisol at biologically inappropriate times. Timing dysregulation may increase long-term neural vulnerability because it undermines recovery periods needed for repair. This disruption may interfere with crucial processes such as, synaptic homeostasis and neuroimmune regulation (Gutierrez Nunez et al., 2025).

Long-term stress can impair glucocorticoid feedback by reducing the sensitivity or functional capacity of feedback loops, often via changes in receptor expression or signaling efficiency. A loss of receptor efficiency in key regions such as the hippocampal and prefrontal circuits can lead to sustained HPA activation, reinforcing a cycle of neuroendocrine pressure (Gutierrez Nunez et al., 2025; Kadmiel and Cidlowski, 2013).

It’s important to note that chronic stress does not always lead to hypercortisolemia. With prolonged exposure, the system may shift towards blunted responsivity. These changes in adrenal and upstream signaling may reduce cortisol reactivity to ACTH or stressors, often resulting in relatively lower output in later stages. This “hypo-reactive” phenotype may reflect compensatory downshifts following chronic overactivation rather than recovery, and it can still be associated with dysfunctional stress regulation and altered immune-endocrine balance (Gutierrez Nunez et al., 2025).

These diverse patterns suggest that chronic stress produces different cortisol profiles depending on the individual and the duration of exposure. Due to this heterogeneity, interpretation of cortisol levels requires caution. To accurately link endocrine changes to cognitive health, researchers must investigate beyond single data points and prioritize more comprehensive interpretations of different metrics, including diurnal rhythms and long-term output (Gutierrez Nunez et al., 2025).

### 3.3 Allostatic load and cumulative neurobiological cost

Allostasis refers to adaptive physiological change in response to stressors, while allostatic load represents the cumulative “wear and tear” that accrues when adaptive systems are repeatedly or persistently activated. The HPA-axis is central to allostatic processes, but chronic activation increases systemic strain by altering metabolic regulation, immune signaling, and neural function (Mbiydzenyuy and Qulu, 2024; Gutierrez Nunez et al., 2025).

Sustained HPA activation and cortisol dysregulation contribute to allostatic load through several interrelated mechanisms. Firstly, prolonged glucocorticoid exposure can disrupt the balance of neurotransmitter systems involved in emotional regulation and stress responsivity, including serotonergic and dopaminergic pathways. Such disturbances can influence affective processing, impulsivity, and behavioral regulation, reflecting stress-driven remodeling within limbic-prefrontal networks (Mbiydzenyuy and Qulu, 2024).

Additionally, chronic neuroendocrine activation may increase vulnerability to stress-related disease states, consistent with evidence linking persistent HPA perturbation to broader physiological dysregulation and immune-mediated pathways. These effects can be amplified when stress exposure co-occurs with psychiatric symptoms, sleep disruption, or lifestyle risk factors, creating a multi-system context that is biologically unfavorable for long-term neural resilience (Gutierrez Nunez et al., 2025).

Furthermore, sustained stress signaling can be associated with structural and functional consequences in stress-sensitive brain regions. The hippocampus and prefrontal cortex are responsible for negative feedback regulation; thus, damage or functional impairment in these regions can further weaken feedback control and drive dysregulation, forming a self-reinforcing loop (Gutierrez Nunez et al., 2025; Herman et al., 2016).

In mechanistic terms, allostatic load provides a framework to explain how repeated endocrine activation can translate into cumulative biological cost, particularly in systems that rely on tight temporal regulation (circadian and ultradian dynamics) and receptor-mediated feedback (GR signaling) (Gutierrez Nunez et al., 2025; Herman et al., 2016).

### 3.4 Glucocorticoid receptor signaling in the brain

Glucocorticoids exert many of their effects through binding to the GR, which is widely expressed across tissues, including the central nervous system. GR signaling influences gene transcription and cellular function across multiple domains, including metabolism, immune regulation, synaptic plasticity, and stress responsivity. Consequently, disruption of GR signaling can produce broad, multi-system consequences relevant to both psychiatric and neurological vulnerability (Kadmiel and Cidlowski, 2013).

The functional impact of GR signaling depends on neuroanatomical context. Evidence from genetic and experimental models indicates that GR activity in forebrain regions plays a key role in regulating HPA-axis feedback and stress-related behaviors, while GR signaling in the amygdala contributes to emotional learning and fear conditioning. These region-specific roles help explain why chronic stress may differentially affect emotional processing, executive control, and memory systems, which are domains frequently implicated in later-life cognitive outcomes (Kadmiel and Cidlowski, 2013).

Chronic stress exposure can alter GR density, sensitivity, and downstream signaling. Reduced GR expression or function in hippocampal and prefrontal regions may weaken feedback inhibition, enabling sustained HPA activation and reinforcing cortisol dysregulation. Additionally, altered GR signaling can interact with immune and inflammatory pathways, contributing to a physiological state in which stress hormones no longer provide appropriate modulation of inflammatory responses. Such dysregulation is particularly relevant in chronic stress contexts where endocrine and immune systems must remain tightly coordinated (Gutierrez Nunez et al., 2025; Kadmiel and Cidlowski, 2013).

Finally, it is essential to distinguish between endogenous glucocorticoids arising from chronic stress and exogenous glucocorticoids. Endogenous cortisol production is tightly regulated, through binding with corticosteroid-binding globulin, which limits free hormone bioavailability, in addition to, tissue-level modulation by 11β-hydroxysteroid dehydrogenase isoforms, which control intracellular glucocorticoid exposure in a region-specific manner. Synthetic glucocorticoids such as dexamethasone or exogenous corticosterone are widely employed in animal stress models and they differ substantially in receptor-binding affinity, resistance to protein binding, tissue penetrance, and metabolic clearance, and can therefore produce biological effects that do not directly mirror those of endogenous cortisol (Kadmiel and Cidlowski, 2013). Where evidence in the following sections derives from exogenous glucocorticoid models rather than chronic stress models, this is noted explicitly, and interpretive caution is applied accordingly.

### 3.5 Aging as a vulnerability amplifier

Aging increases exposure to the harmful neurological effects of chronic stress by altering the biological systems responsible for regulating the stress response (McEwen, 2007). Consequently, older adults are more susceptible to the neurodegenerative effects of prolonged HPA-axis dysregulation. One key factor is the age-related rise in basal cortisol levels observed during human aging, which predicts hippocampal atrophy and memory deficits and reflects a cumulative neuroendocrine burden that compounds over time (Lupien et al., 1998). Moreover, aging is associated with increased neuroimmune reactivity, and the overlapping mechanisms through which stress and aging dysregulate immune function lower the threshold at which chronic cortisol dysregulation triggers neuroinflammation (Attiq et al., 2025; Milligan Armstrong et al., 2021). Preclinical evidence indicates that chronic psychological stress causes neuroendocrine-mediated mitochondrial dysfunction, resulting in compromised neuronal energy production and increased oxidative burden (Picard and McEwen, 2018). These effects are compounded in the aging brain, where mitochondrial bioenergetic capacity progressively declines due to impaired mitochondrial dynamics, reduced mitophagy, and accumulation of mitochondrial DNA mutations (Steffan et al., 2025). Simultaneously, declining proteostasis with age, particularly the progressive weakening of autophagic clearance mechanisms, limits the removal of amyloid and tau aggregates, accelerating their pathological accumulation (Barmaki et al., 2023). Additionally, age-related deterioration of the blood–brain barrier (BBB) further facilitates neuroinflammation by allowing greater immune-cell infiltration into the central nervous system (Yuan et al., 2023). Moreover, reduced neuronal and cognitive reserve decreases the brain’s ability to compensate for stress-related damage, making cognitive impairment more likely (McEwen, 2007; Lucassen et al., 2015).

## 4 Cortisol-induced structural and functional brain alterations

### 4.1 Hippocampal vulnerability and impaired neurogenesis

Among stress-sensitive regions of the brain, the hippocampus appears particularly vulnerable to prolonged cortisol exposure. Postmortem tissue evaluations reveal a wide abundance of GRs tied to the hippocampal area of the brain (Wang et al., 2013). In fact, neuroimaging evidence reveals that chronically elevated cortisol levels are linked to decreased hippocampal volume especially in patients with chronic stress and other mood disorders (Lupien et al., 1998; Moica et al., 2016). Longitudinal MRI studies consistently show that reduced hippocampal volume is strongly associated with memory impairment and predicts progression from Mild Cognitive Impairment (MCI) to AD (Fox et al., 1996; Jack et al., 1999; Petersen et al., 2000). One proposed explanation for this hippocampal shrinkage is that chronic stress and elevated cortisol suppress adult neurogenesis, ultimately leading to structural atrophy and memory decline (Lucassen et al., 2015). While converging neuroimaging and longitudinal evidence supports an association between sustained hypercortisolemia and hippocampal atrophy, causality remains debated due to potential bidirectional interactions between neurodegeneration and HPA-axis dysregulation.

### 4.2 Prefrontal cortex dysfunction and executive impairment

The prefrontal cortex region of the brain subserves many of our cognitive abilities and regulates them. On the other hand, it is also the region most susceptible to the negative consequences of stress exposure (Arnsten, 2009). In animal models, chronic stress induces structural and functional alterations in the prefrontal cortex, characterized by dendritic retraction, spine loss in pyramidal neurons and weakened synaptic connectivity (Czéh et al., 2008; McKlveen et al., 2016; Radley et al., 2006), findings that are consistent with human neuroimaging evidence of prefrontal dysfunction under chronic stress (Arnsten, 2009; Girotti et al., 2018). This disruption in the prefrontal cortex function is associated with impairments in executive processes necessary for goal-directed decision-making while also weakening control over behavior (Girotti et al., 2018). Such dysfunction in the prefrontal cortex region can impair executive functions; however, the extent to which these changes translate into progressive cognitive decline remains unclear.

### 4.3 Amygdala hyperactivation and emotional circuit remodeling

In contrast to hippocampal atrophy, the amygdala exhibits hyperactivation under chronic stress. The amygdala region is rich in GRs making it highly responsive to prolonged cortisol exposure, which increases excitatory activity and emotional memory processing (Myers et al., 2014). In animal models, long-term stress has shown to boost emotional reactivity by increasing dendritic arborization in amygdala neurons, contrasting the reported dendritic atrophy effect of stress on the hippocampus region (Vyas et al., 2002; Vyas et al., 2006). Additionally, several human neuroimaging studies also revealed that stress has a role in altering the structure and function of the amygdala (Tottenham and Sheridan, 2010). Functional neuroimaging research demonstrated increased amygdala activation following stress, indicating increased sensitivity of emotional processing circuits (Tottenham and Sheridan, 2010; van Marle et al., 2010). Such hyper-responsivity in the amygdala may affect the regulation of stress responses, amplifying long-term neurobiological vulnerability (McEwen, 2007; Roozendaal et al., 2009).

### 4.4 Synaptic plasticity disruption

Synaptic plasticity, particularly long-term potentiation (LTP), is a crucial cellular mechanism that serves as the foundation for memory and learning, as LTP at the synapses in the hippocampus and amygdala have shown to support memory consolidation (dos-Santos et al., 2023). However, experimental models indicate that prolonged cortisol elevation has been linked to reduced LTP and may impair this process by altering the synaptic responses and promoting long-term depression (LTD) in hippocampal circuits (dos-Santos et al., 2023; Kim and Diamond, 2002; Xu et al., 1997). Together, these findings indicate that chronic stress can interfere with synaptic mechanisms, ultimately impacting learning and memory.

### 4.5 Excitotoxicity and oxidative stress

Prolonged exposure to high cortisol contributes to neuronal vulnerability through excitotoxic and oxidative pathways. According to Burke et al. (2024), chronic stress and elevated glucocorticoids trigger aberrant glial cell functioning while simultaneously driving glutamate excitotoxicity and over-pruning of synapses. The downstream consequences of this excessive glutamatergic activity, including calcium dysregulation, mitochondrial impairment, and oxidative stress, represent established pathogenic mechanisms spanning both psychiatric disorders and progressive neurodegenerative diseases (Nicosia et al., 2024).

Additionally, chronic cortisol elevation promotes oxidative stress, resulting in cellular damage and neuronal death through various molecular mechanisms, as demonstrated in experimental animal models (Zafir and Banu, 2009) and supported by evidence from human AD pathology (Plascencia-Villa and Perry, 2023). Collectively, these processes can cumulatively damage neurons and increase the risk for neurodegenerative changes that can progress to diseases like AD, as oxidative stress has been strongly linked to AD pathology in literature (Plascencia-Villa and Perry, 2023).

### 4.6 Integrated effects of chronic cortisol on brain structure and function

Chronic cortisol exposure induces contrasting changes in the brain, characterized by hippocampal and prefrontal structural decline alongside amygdala hyperactivation (Arnsten, 2009; McEwen, 2007). This pattern shows that as the prefrontal cortex declines, the brain loses its rational top-down regulatory control and moves toward heightened emotional reactivity driven by the limbic system (Arnsten, 2009; Roozendaal et al., 2009). Impaired hippocampal feedback inhibition of the HPA-axis (Lupien et al., 1998; Lucassen et al., 2015), in addition to declined regulatory control from the prefrontal cortex (Arnsten, 2009), can prolong stress circuit activation. At the same time, the hyper-activation within the amygdala multiplies emotional reactivity, leading to exaggerated responses to stress (Roozendaal et al., 2009).

At the synaptic level, reduced LTP and increased glutamatergic vulnerability impair cell communication (Kim and Diamond, 2002; Nicosia et al., 2024). Along with the neuronal strain from oxidative stress (Plascencia-Villa and Perry, 2023), these factors lower the threshold for network instability. Therefore, sustained cortisol dysregulation may amplify vulnerability, rather than acting as a primary cause, accelerating neurodegenerative susceptibility within stress-sensitive neural circuits (McEwen, 2007; Plascencia-Villa and Perry, 2023).

## 5 Neuroimmune crosstalk and inflammatory amplification

### 5.1 Stress-induced immune dysregulation

The effect of stress on immune function varies with outcome, mainly depending on the duration and severity of biological activation. Acute stress temporarily boosts immune defenses through coordinated neuroendocrine signaling as an adaptive mechanism, while chronic stress exposure disrupts immune homeostasis. Sustained activation of the HPA-axis alters the balance between pro-inflammatory and anti-inflammatory cytokines, leading to chronic low-grade inflammation and impaired immune cell number and function (Dhabhar, 2014). This transition towards immune dysregulation, resulting in systemic inflammation, may compromise the central nervous system (CNS) resilience.

### 5.2 Microglial priming and chronic neuroinflammation

Neuroinflammation has traditionally been defined by overt immune activation within the brain, characterized by microglial activation, increased cytokine and chemokine signaling, infiltration of peripheral immune cells, and localized tissue damage (O’Callaghan et al., 2008). However, modern perspectives recognize that neuroinflammation may also operate silently, leaving brain defenses on constant alert without necessarily manifesting acute inflammatory pathology.

Microglial priming refers to a condition in which microglia exhibit heightened sensitivity to subsequent stimuli following prior stress or inflammatory exposure. In this primed state, even modest secondary insults can trigger exaggerated inflammatory responses. Chronic stress has been implicated in promoting such priming, thereby lowering the threshold for sustained neuroinflammatory activation and promoting neurotoxic signaling cascades within vulnerable neural circuits.

### 5.3 Blood–brain barrier dysfunction

The blood–brain barrier functions not merely as a passive structural barrier, but as a dynamic regulatory interface between systemic circulation and the CNS. Compromise of BBB integrity allows peripheral inflammatory mediators and neurotoxic substances to gain aberrant access to neural tissue, thereby initiating or amplifying inflammatory cascades that would normally be restricted (Yuan et al., 2023).

Beyond structural disruption, dysfunction in BBB transport systems may impair nutrient delivery and toxin clearance, contributing to oxidative stress and metabolic imbalance within the neural microenvironment. Altered endothelial signaling and protein expression increase the production of reactive oxygen species and release inflammatory mediators. This makes the BBB dysfunction a root cause rather than a secondary side effect (Yuan et al., 2023).

### 5.4 Cytokine-glucocorticoid interactions

Glucocorticoid receptors are the principal intracellular mediators of glucocorticoid effects, and their signaling integrity is essential for appropriate physiological and therapeutic responses. However, GR signaling does not operate in isolation; it is embedded within a complex immunoregulatory network influenced by cytokine activity.

Elevated pro-inflammatory cytokine levels, particularly under chronic inflammatory conditions, have been shown to impair GR sensitivity and reduce transcriptional activity (Pace et al., 2007). This cytokine-mediated glucocorticoid resistance may weaken the anti-inflammatory feedback normally exerted by glucocorticoids, thereby perpetuating immune activation. In the context of chronic stress, such dysregulation may create a feed-forward loop in which inflammatory signaling further disrupts endocrine control mechanisms.

### 5.5 Neuroinflammation as a bridge to neurodegeneration

Neuroinflammation arises from a complex interplay between immune signaling and cellular stress responses. Activated glial cells release excessive cytokines and chemokines, promoting recruitment of peripheral immune cells and intensifying local inflammatory activity. Sustained glial activation fosters a self-perpetuating inflammatory cycle characterized by oxidative stress, excitotoxicity, and progressive neuronal injury.

Under prolonged inflammatory conditions, the accumulation of neurotoxic mediators derived from activated glial cells may directly compromise neuronal integrity and synaptic function. When persistent, this inflammatory milieu contributes to structural damage, cognitive impairment, and progression of neurodegenerative pathology (Attiq et al., 2025). Rather than serving as a transient immune response, chronic neuroinflammation may therefore function as a mechanistic bridge linking systemic stress-induced immune dysregulation to long-term neurodegenerative processes.

## 6 Cortisol dysregulation in Alzheimer’s disease pathophysiology

### 6.1 Cortisol and amyloidogenic processing

Chronic activation of the HPA-axis under sustained stress conditions leads to progressive cortisol dysregulation and impaired immune-endocrine balance (Knezevic et al., 2023). Prolonged cortisol elevation has been associated with altered GR sensitivity and downstream transcriptional modulation, mechanisms increasingly implicated in neurodegenerative vulnerability (Knezevic et al., 2023). Clinical observations demonstrate that elevated plasma cortisol levels are associated with accelerated dementia progression in individuals with Alzheimer-type pathology (Csernansky et al., 2006), supporting a temporal relationship between endocrine imbalance and disease advancement. Mechanistic studies provide biological plausibility for cortisol-mediated modulation of amyloidogenic pathways. In neuronal cell and animal models, glucocorticoids have been shown to enhance β-amyloid production via upregulation of BACE1 expression and modulation of *γ*-secretase activity at mitochondria-associated membranes (MAMs) (Choi et al., 2023). *In vivo* experimental models employing exogenous glucocorticoid administration further demonstrate that glucocorticoid exposure increases amyloid-β accumulation and exacerbates tau pathology (Green et al., 2006). These findings suggest that sustained cortisol elevation may influence amyloidogenic processing at both transcriptional and subcellular regulatory levels. Notably, many of these mechanistic studies employ exogenous glucocorticoids rather than chronic psychological stress models; while they establish biological plausibility, translating their findings to endogenous hypercortisolemia warrants caution. Recent population-based neuroimaging data indicate that elevated serum cortisol correlates with increased early amyloid deposition in postmenopausal women (Salardini et al., 2025), suggesting potential sex-specific modulation of cortisol-related amyloid vulnerability. While causality remains complex and potentially bidirectional, converging clinical and experimental evidence supports a mechanistic association between cortisol dysregulation and amyloid pathology.

### 6.2 Tau hyperphosphorylation mechanisms

Tau pathology represents a central correlate of cognitive decline in AD. Evidence from animal models involving exogenous glucocorticoid exposure indicates that chronic glucocorticoid exposure exacerbates tau hyperphosphorylation and accelerates neurofibrillary pathology (Green et al., 2006). These effects may occur through kinase activation, oxidative stress signaling, and impaired synaptic plasticity pathways that are sensitive to glucocorticoid modulation (Knezevic et al., 2023). Importantly, human neuroimaging demonstrates that tau burden correlates more closely with cognitive dysfunction than amyloid deposition across both asymptomatic and advanced stages of clinical disease (Ossenkoppele et al., 2019); meanwhile, experimental models suggest that cortisol-driven tau dysregulation may represent a critical molecular mediator of this progression (Green et al., 2006). Thus, glucocorticoid imbalance may amplify both amyloid-dependent and amyloid-independent neurodegenerative mechanisms.

### 6.3 Mitochondrial dysfunction and metabolic stress

Mitochondrial function is crucial for balanced steroidogenesis. This essential link is evidenced by primary genetic mitochondrial disorders, where cellular dysfunction disrupts hormone synthesis and leads to severe adrenal insufficiency (Corkery-Hayward and Metherell, 2023). Beyond biosynthesis, chronic stress induces structural and functional mitochondrial remodeling, conceptualized as mitochondrial allostatic load (MAL) (Picard and McEwen, 2018). Mitochondrial allostatic load reflects cumulative bioenergetic strain resulting from repeated or sustained stress exposure.

In neural tissue, mitochondrial dysfunction is a well-established feature of AD. Chronic glucocorticoid exposure may exacerbate oxidative stress, impair mitochondrial respiration, and reduce neuronal metabolic resilience (Picard and McEwen, 2018). These processes may converge with amyloidogenic signaling at MAMs (Choi et al., 2023), creating a feed-forward cycle of metabolic stress and proteotoxic accumulation.

### 6.4 Neuroinflammatory amplification and disease acceleration

Elevated cerebrospinal fluid (CSF) cortisol concentrations have been documented in patients with AD and are associated with neuropsychiatric symptom persistence (Ouanes et al., 2022). Chronic HPA-axis activation interacts with immune signaling pathways, promoting microglial activation and sustained neuroinflammatory states (Milligan Armstrong et al., 2021). This interaction may enhance oxidative stress, synaptic dysfunction, and neuronal injury.

Comprehensive mechanistic reviews indicate that chronic stress modulates microglial reactivity and adaptive immune responses, potentially accelerating amyloid and tau accumulation in susceptible models (Milligan Armstrong et al., 2021). As previously noted, cortisol imbalance acts as a multiplier of existing pathologies rather than an independent cause. This notion extends to the cellular level, where it directly accelerates inflammation, mitochondrial decline, and proteostatic dysfunction.

### 6.5 Interaction with genetic susceptibility (e.g., ApoE ε4)

Genetic susceptibility significantly shapes AD risk. The ApoE cascade hypothesis proposes a multistage model in which biochemical alterations in ApoE isoforms lead to cellular dysfunction and eventual phenotypic neurodegeneration (Martens et al., 2022). ApoE4, in particular, promotes impaired lipid metabolism, synaptic instability, and increased amyloid aggregation (Martens et al., 2022).

Within this framework, cortisol dysregulation may further destabilize vulnerable neural systems by intensifying mitochondrial stress and inflammatory signaling (Picard and McEwen, 2018; Milligan Armstrong et al., 2021). Thus, sustained glucocorticoid imbalance may not initiate AD pathology independently but may lower resilience thresholds in genetically predisposed individuals, accelerating disease trajectory.

## 7 Longitudinal and clinical evidence

### 7.1 Prospective cohort studies linking cortisol to cognitive decline

Prospective cohort studies provide temporal evidence linking sustained cortisol dysregulation to progressive cognitive decline. As previously outlined, elevated circulating cortisol levels have been associated with increased memory deterioration over time, alongside measurable hippocampal atrophy in aging populations (Lupien et al., 1998). The duration of cortisol elevation appears to influence the extent of structural and cognitive impairment, supporting a cumulative neuroendocrine burden model.

Prospective cohort evidence further suggests that sustained cortisol dysregulation is associated with accelerated cognitive decline and increased risk of incident dementia in older adults (Ennis et al., 2017; Tsui et al., 2020). Completely ruling out reverse causality is difficult because early neurodegeneration can physically disrupt HPA-axis regulation. However, the longitudinal design of these studies strengthens the likelihood that chronic stress hormone imbalance precedes and actively drives cognitive decline rather than simply reflecting it.

### 7.2 Hair cortisol and long-term exposure biomarkers

Hair cortisol concentration (HCC) has emerged as a biomarker of cumulative long-term glucocorticoid exposure. However, large-scale cohort studies highlight the methodological challenges of isolating its independent effects on brain health. For instance, longitudinal data from the English Longitudinal Study of Aging initially showed an association between elevated hair cortisol and poorer verbal memory, but this link became non-significant in the final adjusted models that controlled for sociodemographic and health-related confounders, including age, wealth, and hypertension. This suggests that confounding factors may account for the observed relationship rather than chronic glucocorticoid exposure alone (Santoso et al., 2022). While HCC captures an averaged record of stress exposure over months rather than temporary fluctuations, these findings indicate that its relationship with cognitive aging cannot be evaluated independently of certain confounding factors.

### 7.3 Stress-related psychiatric disorders and dementia risk

Epidemiological data indicate that clinically diagnosed stress-related disorders are associated with elevated risk of subsequent neurodegenerative disease. A large-scale cohort study reported that individuals with post-traumatic stress disorder (PTSD) and other stress-related conditions exhibited a significantly increased risk of developing dementia, independent of age and major health-related confounders (Song et al., 2020). Notably, risk magnitude correlated with severity and persistence of stress-related pathology, suggesting a potential dose-dependent relationship between chronic stress burden and long-term neurodegenerative outcomes.

Similarly, an independent population-based cohort confirmed that stress-related disorders are associated with heightened dementia risk even after adjustment for familial and medical confounders, reinforcing the robustness of this association (Kim et al., 2023). While psychiatric disorders may represent either risk factors or early manifestations of neurodegeneration, the consistency across cohorts supports the hypothesis that chronic stress exposure contributes meaningfully to long-term brain vulnerability.

### 7.4 Subclinical chronic stress as a silent risk factor

Beyond formal clinical conditions like major depression, hidden chronic stress or constant daily worries have been recognized as silent risk factors for AD and other dementias (Ownby et al., 2006). Chronic activation of the HPA-axis in these populations is frequently accompanied by sustained hypercortisolemia, hippocampal atrophy, and reduced neurogenesis, providing the biological likelihood for the observed epidemiological associations.

In addition, neuroimaging studies of PTSD demonstrate structural and functional brain alterations, including hippocampal volume reduction and network-level dysregulation, patterns that partially overlap with early neurodegenerative changes (Yaffe et al., 2010). While these findings do not establish causality, they suggest that prolonged stress exposure may lower the threshold for neurodegenerative processes, particularly in vulnerable individuals.

### 7.5 Dose–response relationships

Emerging evidence supports a dose–response association between cortisol levels and neurodegenerative progression. Higher plasma cortisol concentrations have been linked to accelerated hippocampal atrophy and increased likelihood of progression from MCI to AD (White et al., 2023). These findings are particularly compelling because they integrate biochemical markers with longitudinal structural imaging outcomes.

Additional studies suggest interactions between cortisol dynamics and sex hormones such as estradiol, potentially modulating stress responsivity and neural vulnerability (Lupien et al., 1998). Individuals with persistently elevated cortisol demonstrate progressive structural brain changes over time. This reinforces the concept mentioned earlier, that sustained glucocorticoid exposure is not a passive symptom of aging, but a biological modifier of disease trajectory that may alter how fast a disease worsens.

### 7.6 Sex differences in stress physiology and Alzheimer’s disease risk

Biological sex is an important but often underappreciated modifier of both the stress response and AD vulnerability. Women account for approximately two-thirds of all AD cases globally, a disparity that cannot be fully explained by longevity differences alone (Podcasy and Epperson, 2016), suggesting that sex-specific biological mechanisms contribute to differential risk. Evidence from the Framingham Heart Study demonstrates that elevated serum cortisol is specifically associated with increased early amyloid deposition in postmenopausal women, suggesting that the hormonal shifts following menopause may amplify the neurotoxic consequences of cortisol dysregulation (Salardini et al., 2025). Recent neuroimaging evidence further supports sex-specific associations between cortisol and AD biomarkers, demonstrating that elevated cortisol levels correlate more strongly with amyloid burden and reduced cerebral glucose metabolism in postmenopausal women compared to men (Alqarni et al., 2026; Mosconi et al., 2024). Notably, this vulnerability is further amplified by ApoE4 carrier status, specifically in women, highlighting a compounding effect of genetic and hormonal risk factors on neurodegenerative susceptibility (Alqarni et al., 2026). Testosterone regulates GR sensitivity and HPA-axis reactivity in men and age-related androgen decline may weaken stress-buffering capacity through different neuroendocrine pathways (Mbiydzenyuy and Qulu, 2024). However the long-term relationship between age-related androgen decline and cortisol-driven neurodegenerative risk in men remains understudied compared to the female evidence base. Given these differences, sex-stratified analyses in future longitudinal and interventional studies will be essential for understanding whether stress-targeted prevention strategies should be tailored differently by sex, particularly in the perimenopausal and postmenopausal context.

## 8 An integrative neuroendocrine-neuroimmune model

The pathways reviewed in preceding sections do not operate independently; instead, they form an interconnected biological network in which chronic stress simultaneously lowers resilience thresholds across multiple systems. HPA-axis dysregulation sustains cortisol dysregulation, which impairs GR-mediated negative feedback and drives further HPA activation. Elevated glucocorticoids concurrently prime microglia toward increased inflammatory reactivity, while cytokine-induced GR resistance weakens the anti-inflammatory modulation that would normally contain this response, creating a feed-forward loop between the endocrine and immune systems (Pace et al., 2007; Milligan Armstrong et al., 2021). Mitochondrial strain and oxidative stress compound synaptic vulnerability while also lowering the threshold for excitotoxic injury and neural network instability (Picard and McEwen, 2018; Plascencia-Villa and Perry, 2023). Together, these stress-related processes speed up amyloid accumulation and tau hyperphosphorylation, worsening existing pathological cascades particularly in individuals already vulnerable due to age, genetic susceptibility, or vascular compromise (Burke et al., 2024; Choi et al., 2023).

Importantly, these pathological processes interact bidirectionally. Downstream consequences of sustained cortisol dysregulation, such as hippocampal atrophy and prefrontal dysfunction, can in turn compromise HPA-axis feedback inhibition, contributing to a cycle of escalating neuroendocrine dysregulation (Lupien et al., 1998; Herman et al., 2016). Similarly, neuroinflammation amplifies proteopathic burden, which in turn sustains glial activation and cytokine release (Attiq et al., 2025).

**Figure 1:**
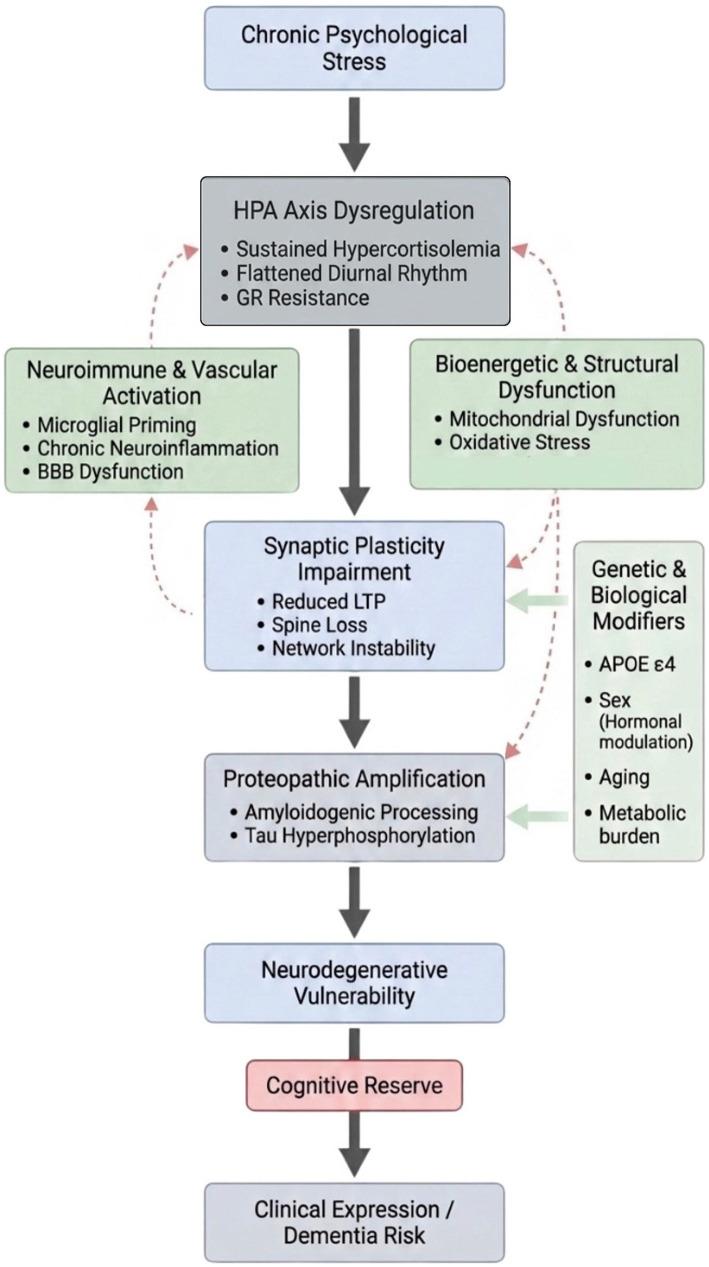
Conceptual diagram illustrating the integrative neuroendocrine-neuroimmune model linking chronic psychological stress to neurodegenerative vulnerability.

Figure 1 illustrates the proposed integrative model linking chronic psychological stress to neurodegenerative vulnerability and AD pathology. The schematic highlights (1) HPA-axis dysregulation as the initiating systemic disturbance, (2) parallel activation of neuroimmune and vascular pathways on one hand, and bioenergetic and structural dysfunction on the other, (3) their convergence at the level of synaptic plasticity impairment, and (4) the resulting proteopathic amplification of amyloid and tau pathology. These central pathways are further shaped by (5) key genetic and biological modifiers, which collectively drive neurodegenerative vulnerability. Bidirectional feedback loops, represented by dashed arrows, illustrate how downstream structural and neuroimmune consequences can in turn amplify HPA-axis dysregulation. The model concludes by demonstrating that the cognitive reserve of an individual acts as a determining threshold, dictating whether this cumulative neural strain translates into the clinical expression of dementia.

## 9 Clinical and translational implications

### 9.1 Chronic stress as a modifiable dementia risk factor

Although chronic stress is not formally classified among established modifiable dementia risk factors in major prevention frameworks (Livingston et al., 2024), converging mechanistic evidence supports its biological plausibility as an upstream vulnerability amplifier. Sustained activation of the HPA-axis results in prolonged glucocorticoid exposure and impaired feedback regulation (Gutierrez Nunez et al., 2025; Herman et al., 2016). Chronic cortisol dysregulation has been associated with structural alterations in stress-sensitive regions such as the hippocampus, a region enriched in GRs (Lupien et al., 1998; Wang et al., 2013), and hippocampal atrophy predicts progression from MCI to AD (Fox et al., 1996; Jack et al., 1999).

Elevated long-term cortisol exposure has also been linked to increased risk of AD in prospective cohorts (Ennis et al., 2017), while meta-analytic data indicate cortisol hypersecretion in patients with AD compared with cognitively unimpaired controls (Zheng et al., 2020). However, as noted previously, the current body of evidence remains predominantly observational, and reverse causality, where early neurodegeneration disrupts HPA-axis regulation, cannot be excluded (Zheng et al., 2020). Thus, formal classification of chronic stress as a modifiable dementia risk factor requires prospective interventional validation.

Notably, these insights are particularly significant for older populations, given that dementia incidence rises steeply with age and the biological vulnerabilities that amplify stress-related neurodegenerative risk. Specifically, these include declining HPA-axis feedback efficiency, reduced neuronal reserve, and heightened neuroimmune reactivity (McEwen, 2007).

### 9.2 Early identification of HPA-axis dysregulation

Early identification of sustained HPA-axis dysregulation may offer translational relevance for dementia risk stratification. Elevated circulating cortisol levels have been associated with hippocampal atrophy and memory decline in aging populations (Lupien et al., 1998). Morning serum cortisol sampling (typically between 7:00–9:00 a.m.) captures peak circadian secretion (Uchoa et al., 2014), while salivary cortisol assessment enables evaluation of diurnal slope and cortisol awakening response, reflecting dynamic free cortisol regulation (Stalder et al., 2016). Neuroimaging modalities such as MRI detect structural alterations in stress-sensitive regions, particularly hippocampal atrophy, which predicts progression to AD (Fox et al., 1996; Jack et al., 1999). Nevertheless, heterogeneity in cortisol measurement techniques and methodological variability across studies limit standardization and immediate clinical implementation (Zheng et al., 2020).

### 9.3 Preventive and lifestyle interventions

Given the mechanistic links between chronic stress, HPA-axis dysregulation, and neurodegenerative vulnerability (Gutierrez Nunez et al., 2025; Knezevic et al., 2023), stress-reduction strategies may represent a complementary component within multidomain dementia prevention models. Psychological stress has been associated with increased dementia risk in large population-based cohorts (Song et al., 2020; Kim et al., 2023), and stress-related psychiatric conditions such as depression are linked to higher AD risk (Ownby et al., 2006). These findings support the hypothesis that reducing chronic stress burden may mitigate downstream neuroendocrine and inflammatory dysregulation. Chronic stress is known to promote microglial activation and neuroinflammatory amplification (Milligan Armstrong et al., 2021), as well as mitochondrial dysfunction conceptualized as MAL (Picard and McEwen, 2018), both of which are implicated in AD pathophysiology (Attiq et al., 2025).

However, while observational and mechanistic data are compelling, robust randomized interventional trials demonstrating that stress-reduction directly alters dementia incidence remain limited. Thus, stress-targeted interventions should currently be considered biologically plausible but not yet causally established preventive strategies.

### 9.4 Pharmacological targeting of glucocorticoid Signaling

Pharmacological modulation of glucocorticoid signaling represents a potential translational avenue, given the role of GR pathways in regulating neuroendocrine and immune interactions (Kadmiel and Cidlowski, 2013). Chronic cortisol elevation has been implicated in amyloidogenic processing and tau hyperphosphorylation in preclinical models (Choi et al., 2023; Green et al., 2006), and elevated cortisol levels are associated with accelerated dementia progression in clinical cohorts (Csernansky et al., 2006).

Furthermore, cortisol dysregulation interacts with neuroinflammatory mechanisms (Milligan Armstrong et al., 2021) and may impair mitochondrial function under sustained stress exposure (Picard and McEwen, 2018). These converging pathways suggest that therapeutic strategies targeting GR signaling or downstream endocrine–immune interactions may modify disease trajectories. Nevertheless, given the systemic physiological roles of glucocorticoids (Herman et al., 2016), indiscriminate suppression of cortisol signaling may produce adverse metabolic and immunological consequences. Therefore, precision-based modulation rather than global inhibition would likely be required, pending validation in controlled clinical trials.

### 9.5 Public health implications

Chronic psychological stress is highly prevalent and frequently co-occurs with socioeconomic adversity and psychiatric morbidity, both of which are associated with increased dementia risk (Song et al., 2020; Kim et al., 2023). From a public health perspective, stress may therefore function as a population-level vulnerability amplifier rather than an isolated risk factor. Emerging evidence supports a dose–response relationship between cortisol levels and hippocampal atrophy as well as progression from MCI to AD (White et al., 2023), suggesting that cumulative neuroendocrine burden may influence disease trajectory. Integration of stress biology into dementia prevention frameworks could expand current models beyond vascular and metabolic determinants (Livingston et al., 2024), incorporating neuroendocrine resilience as an additional dimension of risk stratification. However, policy translation requires stronger longitudinal and interventional evidence to determine whether modifying chronic stress exposure meaningfully reduces dementia incidence.

## 10 Discussion

Translating cellular mechanisms into clinical practice is crucial for understanding the relationship between the strain caused by chronic stress and neurodegenerative vulnerability. This section addresses existing methodological limitations in the literature, and outlines future directions.

### 10.1 Methodological limitations in the current literature

Current evidence linking chronic stress, cortisol dysregulation, and neurodegenerative vulnerability is subject to several important methodological limitations. A major challenge concerns heterogeneity in cortisol assessment. Across studies, cortisol has been measured using serum, salivary, and hair samples, each capturing distinct biological dimensions such as single time-point levels, diurnal slope patterns, or cumulative long-term exposure. Variability in sampling timing, assay techniques, adherence to collection protocols, and operational definitions of “HPA-axis dysregulation” complicates cross-study comparability and limits reproducibility. Although HCC provides a promising index of chronic glucocorticoid exposure, it has not been consistently implemented across longitudinal cohorts, and standardized reference ranges remain insufficiently established.

A second critical limitation relates to reverse causality and bidirectional interactions. While chronic hypercortisolemia has been associated with hippocampal atrophy, amyloid deposition, and tau hyperphosphorylation in both clinical and experimental contexts, early neurodegenerative changes may themselves disrupt stress-regulatory circuits and impair feedback inhibition of the HPA-axis. This temporal ambiguity complicates causal inference and prevents definitive conclusions regarding whether sustained cortisol elevation represents a primary driver of neurodegeneration or a downstream consequence of evolving pathology.

Furthermore, chronic HPA-axis activation frequently coexists with psychiatric disorders, sleep disturbances, metabolic dysregulation, and adverse lifestyle factors, many of which independently increase dementia risk. Stress-related conditions such as PTSD and major depressive disorder are associated with elevated dementia incidence, raising the possibility of residual confounding and shared vulnerability pathways. Disentangling the independent contribution of cortisol dysregulation from these overlapping determinants remains challenging, particularly in observational study designs where stress exposure clusters with socioeconomic and behavioral risk factors.

Importantly, much of the current literature derives from observational cohorts, neuroimaging studies, and animal models that primarily demonstrate associations rather than causality. Randomized controlled trials evaluating whether stress reduction or targeted modulation of glucocorticoid signaling alters long-term neurodegenerative trajectories remain scarce. The limited availability of interventional evidence restricts the ability to confirm a causal role for sustained cortisol elevation in dementia development.

Finally, translational inconsistency persists between experimental and clinical findings. Animal models demonstrate that prolonged glucocorticoid exposure can exacerbate tau pathology and accelerate neurodegenerative cascades; however, translating these mechanistic insights into human populations is complicated by species-specific neurobiology, differences in stress paradigms, and variability in chronic exposure patterns. Similarly, neuroimaging associations between hypercortisolemia and structural brain atrophy do not necessarily establish direct molecular equivalence with experimental findings. A further translational limitation concerns the distinction between endogenous cortisol elevations arising from chronic psychological stress and the exogenous glucocorticoid models employed in many mechanistic studies; differences in potency, bioavailability, and receptor dynamics between these contexts limit the directness of translation to human chronic stress physiology. Addressing these limitations will require harmonized cortisol measurement protocols, longitudinal multimodal biomarker integration, and carefully designed interventional trials to clarify causal pathways and clinical applicability.

### 10.2 Future directions

Cortisol dysregulation should not be reduced to simply high or low cortisol levels, as the imbalance in its regulation can manifest in several different physiological patterns. These may include hypercotisolemia, a flattened diurnal slope, impaired feedback, or, in later stages, a hyporeactive phenotype. Therefore, treating all these patterns as a single form of cortisol dysregulation is a very simplistic concept that does not reflect the true complexity of the HPA-axis. Consequently, it is crucial to understand that cortisol dysregulation does not pose the same risk to everyone, as each pattern can be associated with different effects on the brain and cognition. For this reason, more accurate individual profiles are important, taking into account measurements, duration of chronic stress, receptor sensitivity, and factors such as age, sex, and genetic predisposition.

Cortisol levels alone are inadequate for assessing the comprehensive biological effects of chronic stress, mainly due to methodological discrepancies among studies and numerous ways of measuring employed, such as the use of saliva, serum, and hair cortisol analysis. Therefore, it is necessary to integrate biomarkers to combine cortisol measurements with imaging findings such as MRI, as well as BBB disturbances and inflammatory markers. However, this approach may help reduce the weakness in causality, and developing standardized measurement protocols will be crucial for improving future studies.

The main issue currently is that most evidence is observational and not causal. Therefore, future research must incorporate evidence from randomized longitudinal trials to determine whether reducing stress or modifying glucocorticoid signaling can successfully slow cognitive decline. These studies should focus on measurements other than cortisol, such as hippocampal volume, the rate of conversion from MCI to AD, and possibly amyloid/tau biomarkers.

Additionally, a translational gap remains when applying animal data to humans, thereby making it essential to develop models that can precisely reflect human conditions. Consequently, it is crucial to develop animal models that accurately represent realistic patterns of chronic exposure, while accounting for variations in biological circadian cycles and the interplay between stress, inflammation, and tau and amyloid diseases. When the results of animal studies are linked to human indicators such as neuroimaging indicators and long-term cortisol measurement models, the interpretive value will be enhanced.

Furthermore, chronic stress could affect neurodegeneration; however, it has not been established as a direct causal preventive target for dementia. Consequently, future efforts should incorporate stress management in dementia prevention, encompassing psychological support, stress reduction techniques, enhancement of sleep quality, lifestyle modifications, and potentially strategies aimed at regulating the HPA-axis. It is important to incorporate these strategies into multidomain prevention frameworks and preventative models instead of considering them as isolated interventions.

## 11 Conclusion

In summary, our mechanistic review demonstrates that cumulative stress exposure disrupts the HPA-axis, producing neuroendocrine imbalance. Chronic activation of GRs allows allostatic load to accumulate within vulnerable neural structures, simultaneously, impairing cerebral plasticity and driving oxidative damage. This combined cellular strain acts as an amplifier of progressive impairment across broader neural networks related to memory and executive control. Consequently, this sustained physiological stress activates downstream immune pathways, contributing to microglial priming, BBB breakdown, and cytokine-induced alteration of GR signaling. While this chronic cortisol dysregulation does not independently initiate AD, it actively amplifies the severity of existing pathological processes, including amyloid deposition, hyperphosphorylation of tau protein, mitochondrial dysfunction, and oxidative stress, which collectively accelerate neuroinflammation. Ultimately, targeting prolonged glucocorticoid exposure remains a key strategy for preserving structural brain health and preventing neurodegenerative decline in aging populations.
